# Mortality due to respiratory system disease and lung cancer among female workers exposed to chrysotile in Eastern China: A cross-sectional study

**DOI:** 10.3389/fonc.2022.928839

**Published:** 2022-08-01

**Authors:** Zhaoqiang Jiang, Junqiang Chen, Junfei Chen, Lingfang Feng, Mingying Jin, Huixian Zhong, Li Ju, Lijin Zhu, Yun Xiao, Zhenyu Jia, Chengmin Xu, Difang Yu, Xing Zhang, Jianlin Lou

**Affiliations:** ^1^ School of Public Health, Hangzhou Medical College, Hangzhou, China; ^2^ Emergency Management Bureau of Hangzhou Bay New Area, Ningbo, China; ^3^ School of Medicine, and The First Affiliated Hospital, Huzhou University, Huzhou, China

**Keywords:** asbestos, cause of death, mortality, lung cancer, mesothelioma

## Abstract

Female workers in the asbestos processing industry of Eastern China are at high risk of developing multiple types of cancer, and more data are urgently needed to better understand and address this issue. Death certificate data were selected from an asbestos processing city in China from 2005 to 2006. Information was investigated using the relatives of those individuals who had died as sources of information. Individuals were classified into one of three asbestos exposure levels. Standardized mortality ratio and 95% confidence interval were calculated. A total of 2,964 individual deaths were identified from 2005 to 2006; of these, 21.4% were occupationally exposed to asbestos. The main cause of death was circulatory system diseases (21.2%). The proportion of individuals with respiratory system diseases increased by age among each exposure subgroup (*P*_trend_ < 0.01). Among females, a significant trend was observed between increased asbestos exposure and mortality due to respiratory system diseases and lung cancer. Our study indicated that asbestos exposure was associated with excess mortality from lung cancer and respiratory diseases, particularly among female workers in an asbestos processing area in Eastern China.

## Introduction

Asbestos is comprised of a set of naturally occurring silicate minerals. Historically, was widely used in the construction and manufacturing industries. China currently is the largest asbestos consumer, and its annual asbestos production is among the top three producing countries (1). Chrysotile—a major type of asbestos—has been widely used in the asbestos processing industry of Eastern China since the 1960s (2).

Asbestos is known to cause serious respiratory effects such as asbestosis, pleural diseases, lung cancer, and mesothelioma; collectively, these are known as asbestos-related diseases (3). As a result, the International Agency for Research on Cancer (IARC) and many countries have strictly controlled asbestos as a statutory carcinogen (4). According to World Health Organization (WHO) database results, from 1994 to 2010, the global burden of asbestos-related diseases was substantial, with average potential years of life lost of 17 and 13 years for mesothelioma and asbestosis, respectively (5). Since the 1970s, the movement towards a global ban on asbestos began, as many developed countries banned its use (6). Unfortunately, asbestos is still largely used in many developing countries like China and India. As a result, the threat of asbestos-related diseases is still present for a large number of asbestos-exposed workers (7). Critically, there has been under-reporting of actual cases of asbestos-related diseases in many developing countries, including China (8).

Although a few studies indicate no excess mortality from lung cancer among asbestos-exposed workers (9), many more epidemiological studies have found excess mortalities from lung cancer and mesothelioma among asbestos-exposed workers in developed countries like Italy and Japan (10, 11). A significantly increased standardized mortality ratio (SMR) for asbestosis (SMR = 368.05) was also observed in asbestos-exposed workers in Italy. Moreover, data from a cohort of 577 chrysotile-exposed workers in West China showed that asbestos exposure was associated with excess mortality from lung cancer and respiratory diseases (12). Similar trends for lung cancer, gastrointestinal cancer, nonmalignant respiratory diseases and all cancers were found in a cohort of people who worked in the chrysotile mines in Northwest China (13). However, all of the asbestos-exposed workers in this cohort were male; comparatively, other industries have a higher percentage of female workers. To this end, past work has shown a large female-to-male ratio of workers in the asbestos processing industry of Eastern China (14). This results in a large difference in work and lifestyles between Western and Eastern China. Furthermore, few studies have conducted a face-to-face investigation to analyze the causes of death in a population with a strong history of asbestos exposure. To this end, a 40-year cohort study among female, hand-spinning chrysotile workers in China showed that SMRs for total cancer and lung cancer deaths were elevated (15).

Estimates have suggested that prevalence, incidence, and mortality due to asbestos-related diseases would be expected to be high. Moreover, they would increase for decades, owing to historically high volumes of asbestos used coupled with a long latency period of asbestos-related cancers (16). Hence, it was of great importance to assess the cause of death among chrysotile-exposed workers in Eastern China. This was done to understand the causes of death and their proportions in asbestos-exposed workers. Ultimately, this will provide new insights into the etiology of causes of death after asbestos exposure.

## Material and methods

### Study area

The study area was located in an important asbestos processing and export city in Eastern China. There was no chrysotile mine in the study city. The raw material for processing was chrysotile, which had been obtained from West China, Northwest China, Russia, and Canada. The asbestos processing industry of this city began in the 1950s and peaked from 1970 to 1980. During this time, 33,585 workers accumulated in the asbestos processing industry of this city; from 1996 and beyond, this population decreased (17). Due to the industrial restructuring of the 1990s, most asbestos processing factories closed down. According to unpublicized data from the local government, the annual asbestos processing capacity per year was as follows (in tons): 4200 ± 300 from 1960 to 1969, 6300 ± 500 from 1970 to 1979, 7800 ± 200 from 1980 to 1989, 5500 ± 200 from 1990 to 1999, and 3000 ± 200 from 2000 to 2009. We analyzed the asbestos purity in three samples of Russian asbestos sources and 13 samples of Chinese asbestos sources in our previous study (18). The results using the X-ray diffraction method confirmed that the asbestos sources from asbestos processing factories were all chrysotile, but some were mixed with SiO_2_, CaCO_3_, and other impurities. In the study city, we selected six typical districts out of 19 total districts. Manufacturing—including manufacturing electrical machinery and equipment, chemical fibers, and general equipment—was the main industry of the city. There were 1.02 million residents in the study city in 2005, while the mean population in 2005 and 2006 in the studying districts was 0.4 million (39.2%).

### Questionnaire surveillance

A household survey was conducted among all relatives of those who had died during the study period. The survey was fielded from Feb. 1, 2007 to Jun. 31, 2007. Main caregivers such as sisters, brothers, and spouses of the individual who died were selected as respondents. **Supplementary Information** was obtained from other family members of those who had died. A self-designed structured questionnaire was used for data collection. This questionnaire contained questions on the sociodemographic profile, smoking history, asbestos-related job, and domestic asbestos exposure of the individual who had died. All investigators were thoroughly trained, with a focus on the questionnaire contents and appropriate communication approaches when interacting with respondents. After training, a face-to-face interview was conducted for survey data collection.

### Definition of asbestos exposure by district

According to our previous investigation (unpublished data), we subjectively classified six districts into three asbestos exposure groups: (1) Low asbestos exposure (district was far from the asbestos processing area and this district itself did not have any asbestos processing plants), (2) medium asbestos exposure (district had< 10 plants), and (3) high asbestos exposure (district had ≥ 50 asbestos processing plants).

### Definition of individual asbestos exposure

The duration of occupational exposure to asbestos was defined as the interval between the starting and ending years of asbestos processing. Based on collected information regarding the job, exposure duration, and domestic exposure to asbestos, we defined individual asbestos exposure levels as follows: (a) low asbestos exposure, where the individual had been engaged in an asbestos-related job for<1 year and/or there was no asbestos processing plant near the individual’s residence (> 1000 m), (b) medium asbestos exposure, where the individual had been engaged in an asbestos-related job for 1-10 years, and (c) high asbestos exposure, where the individual had been engaged in an asbestos-related job for 10+ years. We defined non-occupational exposure to asbestos as no asbestos exposure from any asbestos-related job and/or an asbestos processing plant near the individual’s residence (< 1000 m).

### Mortality data

Considering the incubation period of asbestos-induced lung cancer, we obtained a registration database of injury and death certificates registered from Jan. 1, 2005 to Nov. 31, 2006 from local official departments. The address of death cases in these records was specified as the study city. Cases whose ages at death were ≥ 30 were included in the study. Causes of death were coded according to the International Classification of Diseases 10th Revision (ICD10). Causes of death were further classified into one of nine categories: Respiratory system diseases, circulatory system diseases, lung cancer, liver cancer, gastric cancer, esophageal cancer, cancers of the colon, rectum, and anus, and other malignant tumors, and other causes of death. In our study, respiratory system diseases were defined as tuberculosis, chronic respiratory diseases, pneumonia, and other benign diseases of the respiratory system. Other malignant tumors included leukemia, cervical carcinoma, nasopharyngeal carcinoma, bladder cancer, mammary cancer, and other malignant tumors. Other causes of death included accidents, being killed, and suicide among others. Initially, we identified 3,073 individuals in the registration database; of these, 109 were excluded as we could not contact their relatives. A total of 2,964 individuals were included in our study.

### Ethical considerations

The current study was conducted in accordance with the Declaration of Helsinki and was approved by the Ethics Committee of the Zhejiang Academy of Medical Sciences, China. All relatives of those individuals who had died and were included in the study provided informed consent and agreed to participate in this surveillance.

### Statistical analysis

Age was presented as ( $\bar{x}\pm s_{e}$ ). Qualitative data were presented as frequency and proportion distributions. Either Chi-square statistic or Fisher exact test was used for testing associations in the data; pairwise comparisons were made with the Bonferroni method adjusted P-values. Cochran-Armitage trend test was used to test if the proportion of lung cancer or respiratory system diseases had a significant trend over time. Standard proportional mortality ratio (SPMR) and 95% confidence interval were calculated based on comparison with local, historical population mortality data from the study city from 2005 to 2006 and mortality data of Eastern China in 2006, respectively (19). Two-side P< 0.05 was considered statistically significant. R software (version 3.5.0) was used for all statistical analyses.

## Results

### Social demographic characteristics and causes of death

Among the 2,964 individuals who had died and were identified in the study city, 1,144 (38.6%) were women (**Table 1**). The youngest and oldest ages at the time of death were 30 and 79, respectively. Of those who had died, 46.5% were smokers and 21.4% were occupationally exposed to asbestos. The district with high levels of asbestos exposure had 45.5% of all deaths from 2005 to 2006. The main cause of death from 2005 to 2006 was circulatory system diseases, which was the cause of death in 21.2% of all included individuals. Respiratory system diseases and lung cancer accounted for 17.3% and 12.6%, respectively. Three cases of larynx cancer and four cases of ovary cancer were detected.

**Table 1:** Social demographic characteristics and causes of death among the study population.

Index	Statistics [*n* (%)]
Age	65.4 ± 12.0^*^
Gender	
Male	1820 (61.4)
Female	1144 (38.6)
Smoking status	
No	1436 (48.4)
Sometimes	150 (5.1)
Always	1378 (46.5)
Individual occupational exposure to asbestos	
Low	2328 (78.5)
Medium	395 (13.3)
High	241 (8.1)
Non-occupational exposure to asbestos	
No	2359 (79.5)
Yes	605 (20.4)
Asbestos exposure level by districts	
Low	939 (31.7)
Medium	675 (22.8)
High	1350 (45.5)
Causes of death	
Lung cancer	413 (13.9)
Liver cancer	372 (12.6)
Gastric cancer	208 (7.0)
Esophageal cancer	59 (2.0)
Cancer of colon, rectum and anus	38 (1.3)
Other malignant tumors	219 (7.4)
Respiratory system diseases	512 (17.3)
Circulatory system diseases	629 (21.2)
Other causes	514 (17.3)

* displayed as mean ± standard deviation.

### Females had higher asbestos exposure levels

**Figure 1:**
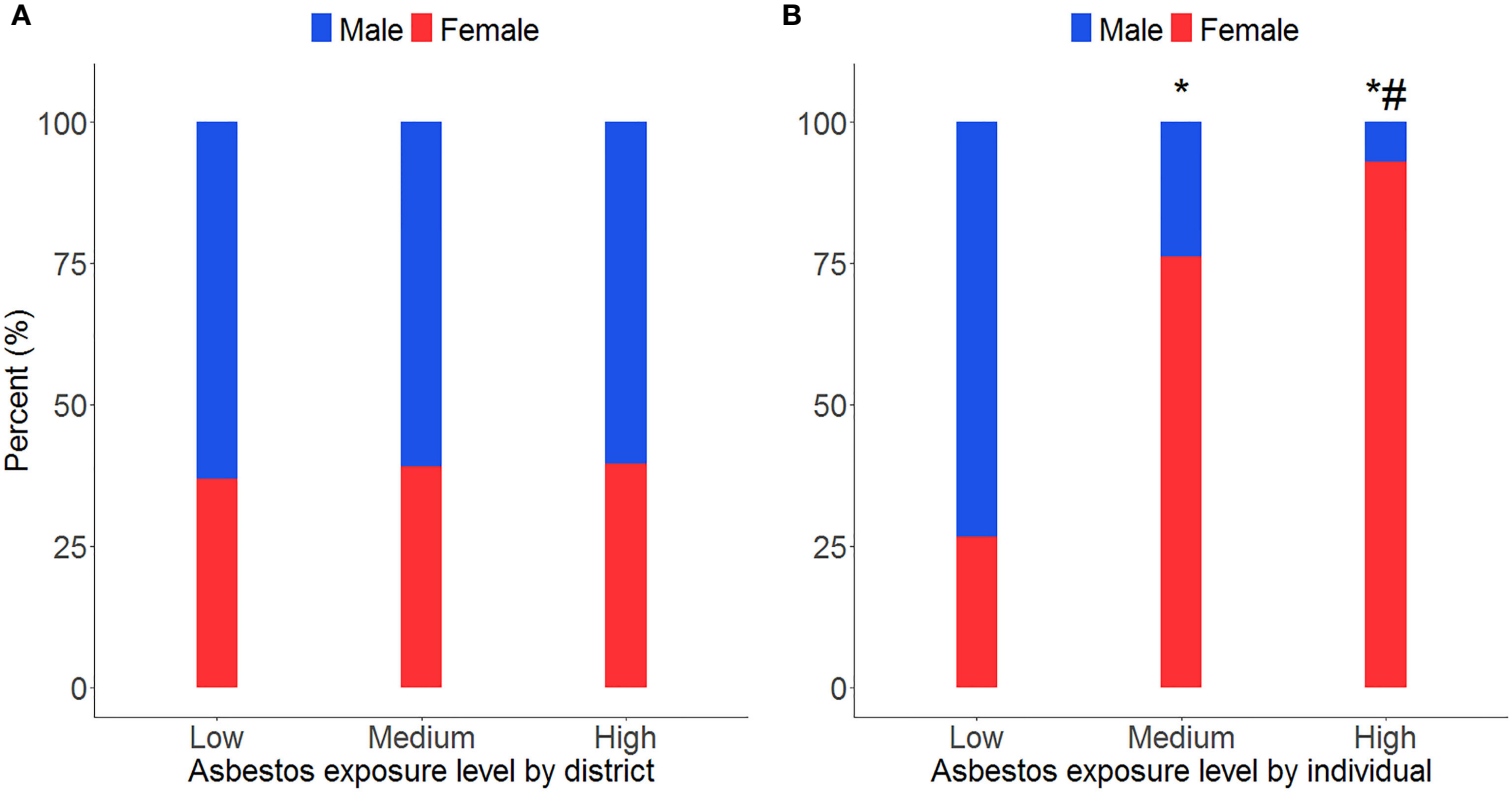
Asbestos exposure duration by district and gender. *P< 0.01, compared with males; **(A)** compared with individuals in districts with low density of asbestos processing plants, *P<* 0.01; **(B)** compared with individuals in districts with the medium density of asbestos processing plants, *P<* 0.01.

When separating individuals by their district’s asbestos exposure level, there was no significant difference in gender distribution among the different groups (χ^2^ = 0.18, P = 0.91; **Figure 1A**). However, when dividing individuals by individual asbestos exposure level, results indicated a difference across gender between different groups (χ^2^ = 105.03, P< 0.01; **Figure 1B**). More specifically, the proportion of females in the medium and high asbestos exposure groups was much higher (P< 0.01) than that in the low asbestos exposure group. Of those who had medium levels of exposure to asbestos, most (76.2%) were female and only 23.8% were male. Comparatively, most individuals in the low asbestos exposure group were male (73.4%) and only 26.6% were female. Notably, the female proportion (92.9%) was highest in the group with high asbestos exposure levels when compared with the proportion in the medium and low exposure groups.

### The proportion of individuals with respiratory system diseases increases with age

**Figure 2:**
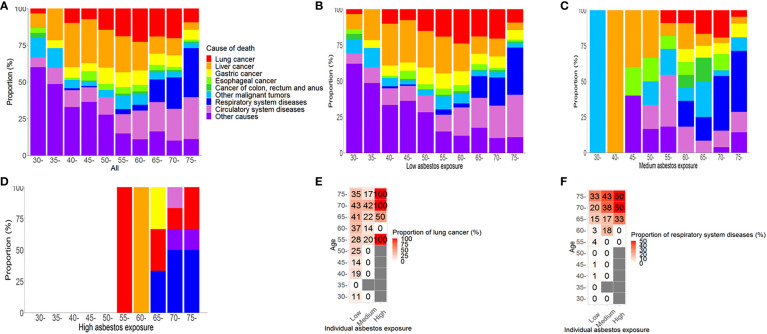
Causes of death by asbestos exposure level and age group among males. **(A)** Proportions of causes of death in all males; **(B)** Proportions of causes of death among males with low asbestos exposure; **(C)** Proportions of causes of death among males with medium asbestos exposure g; **(D)** Proportions of causes of death among males with high asbestos exposure; **(E)** Proportion of individuals who died from lung cancer in subgroups of each cross table among males; **(F)** Proportion of individuals who died from respiratory system disease in subgroups of each cross table among males.

**Figure 3:**
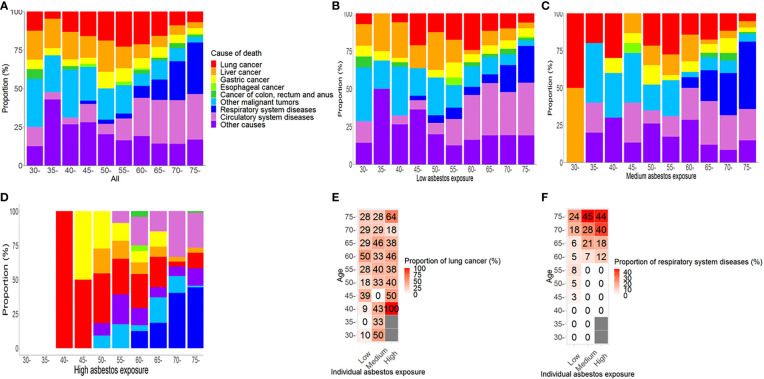
Causes of death by asbestos exposure level and age group among females. **(A)** Proportions of causes of death in all females; **(B)** Proportions of causes of death among females with low asbestos exposure; **(C)** Proportions of causes of death among females with medium asbestos exposure; **(D)** Proportions of causes of death among females with high asbestos exposure; **(E)** Proportion of individuals who died from lung cancer in subgroups of each cross table among females; **(F)** Proportion of individuals who died from respiratory system disease in subgroups of each cross table among females.

In males (**Figure 2**) and females (**Figure 3**), the proportion of causes of death changed by asbestos exposure level and age group. Within each asbestos exposure group, the proportion of those who died from respiratory system diseases increased by age among males (Z = -6.42, P< 0.01; **Figures 2A-D**) and females (Z = -4.89, P< 0.01; **Figures 3A-D**). However, deaths from lung cancer were not related to age in either males (Z = -0.66, P = 0.51) or females (Z = -1.68, P = 0.09). In the high asbestos exposure group, the highest proportion of lung cancer (100%) was in individuals aged 55-59, but only because there was a single case of lung cancer in this subgroup. Among the high asbestos exposure group, 33% of males over 75 years died of lung cancer, while 44% of females over 75 years did. Detailed proportions of deaths due to lung cancer and respiratory system diseases are shown below among males (**Figures 2E, F**) and females (**Figures 3E, F**).

### Mortality by gender and individual asbestos exposure

**Figure 4:**
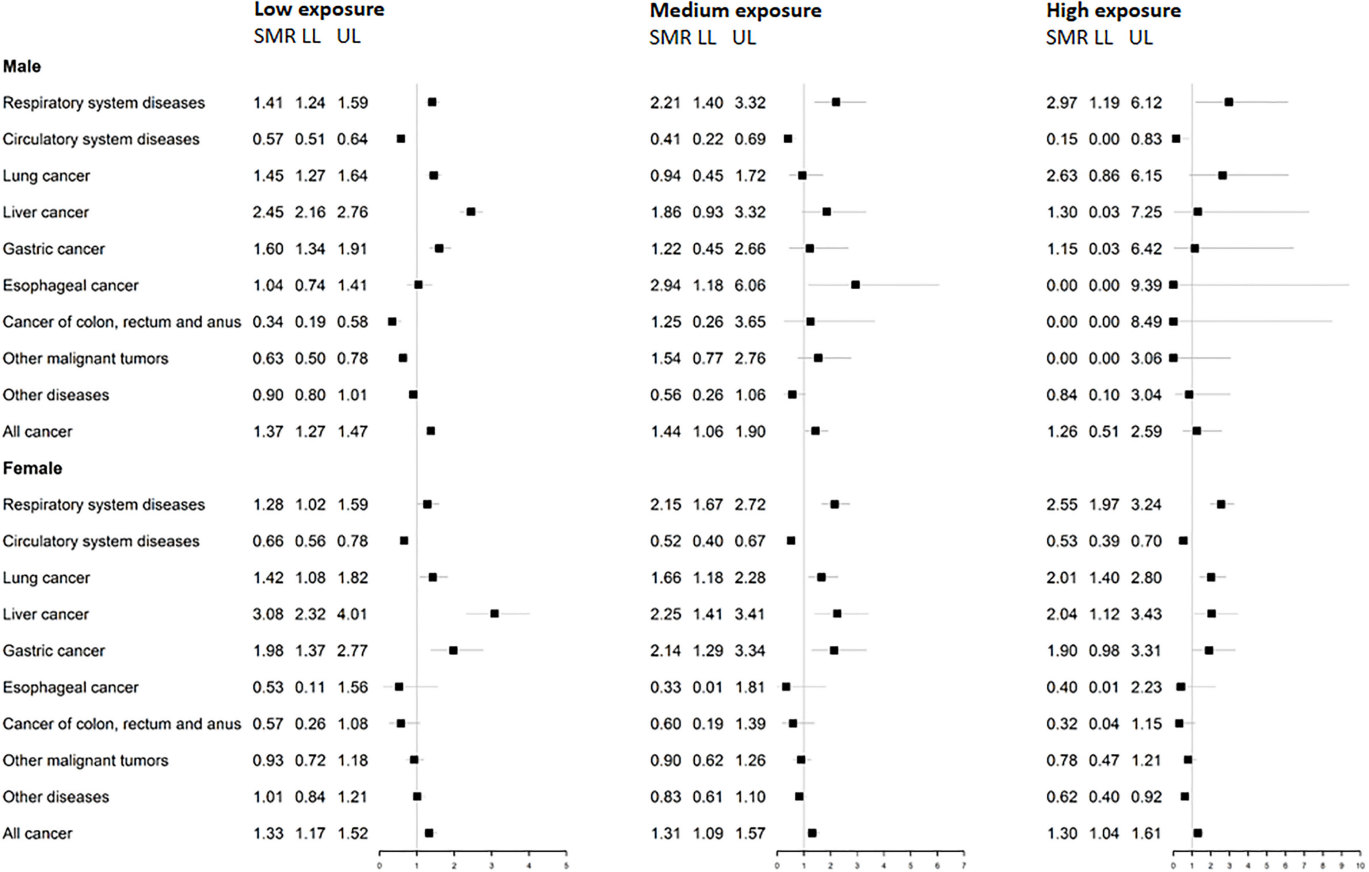
SPMRs and 95% CIs compared with mortality data of Eastern China by gender and district of different asbestos exposure level. SPMRs and 95% CIs were stratified by asbestos exposure levels; *LL*, lower limit of the confidence interval; *UL*, upper limit of the confidence interval.

Compared with the population in Eastern China, males in the study area had excess mortality due to respiratory system disease among low, medium, and high asbestos exposure levels (SPMR = 1.41, 2.21, and 2.97, respectively; **Figure 4**). Excess mortality due to lung cancer was found among males who were not exposed to asbestos (SPMR = 1.45). Mortality due to the circulatory system diseases in males was significantly lower than that in Eastern China’s general population (SPMR = 0.57, 0.41, and 0.15, respectively). Mortality due to liver and gastric cancers was the highest among males with low asbestos exposure (SPMR = 2.45, and 1.6, respectively). When compared with the general population in Eastern China, males had the highest mortality in the medium asbestos exposure level (SPMR = 2.94). Observed and expected deaths of the studied population were provided (**Supplementary Table 1**).

Among females, SPMR from respiratory system disease was 1.28, 2.15, and 2.55, respectively among low, medium, and high exposure groups (**Figure 4**). Mortality due to circulatory system diseases was reduced among females from each asbestos exposure level (SPMR = 0.65, 0.52, and 0.53, respectively). Notably, a significant, dose-dependent relationship was found between asbestos exposure level and lung cancer mortality among females (SPMR = 1.42, 1.66, and 2.01, respectively). Liver cancer mortality was elevated among females of each asbestos exposure level (SPMR = 3.08, 2.25, and 2.04, respectively). Deaths due to gastric cancer among low and medium exposure groups showed excessive mortality compared with the general population of Eastern China (SPMR = 1.98, and 2.14, respectively). All cancer mortality was also elevated at each asbestos exposure level (SPMR = 1.33, 1.31, and 1.30, respectively).

## Discussion

The proportion of deaths where individuals had some asbestos exposure was more than 50% in most of the study city’s districts. Consequently, a high proportion of deaths due to lung cancer and respiratory system diseases was observed among individuals with asbestos exposure. Furthermore, we found that the high proportion of females was related to a high level of asbestos exposure duration and potential exposure level to asbestos. An elevated trend was observed between asbestos exposure levels and mortality due to respiratory system diseases and lung cancer among females. The association between mortality due to respiratory system diseases and lung cancer and asbestos exposure was robust—it did not matter if we used local mortality data or mortality data for the general population of Eastern China. Collectively, these results indicated that excess mortality from respiratory system diseases and lung cancer was strongly associated with asbestos exposure among females.

We found a large proportion of females engaged in the asbestos processing industry in the study area. This finding agreed with previous work from Eastern China (20) but differed from studies in Western countries (21). There was a greater proportion of females in the medium and high asbestos exposure groups than in those who had low levels of asbestos exposure. This result is explained by the fact that hand-spinning chrysotile was the main asbestos processing industry in this study area and that women were deemed more suitable for asbestos textile jobs than men. In fact, thousands of women participated in asbestos processing jobs from the 1950s to the 1990s, resulting in a long duration of asbestos exposure among women. Given this, the long-term exposure to asbestos among women requires a high degree of attention in this study area.

Circulatory system diseases, lung cancer, and respiratory system diseases were the leading causes of disease-related deaths in the study area. The age-standardized death rate of cardiovascular diseases was the highest among all causes of death in China, which was 307.18 per 100,000 people in 2013 (22). We found lower mortality due to circulatory system diseases in females across all asbestos exposure groups. This result was consistent with previous work (23) in Italy, which reported a reduced risk of cardiovascular diseases (SPMR = 0.57, 95% CI = 0.48–0.68) among workers in an asbestos cement factory. Other studies (24, 25) have also reported reduced mortality due to circulatory diseases among workers exposed to either chrysotile or amphibole. However, the association between asbestos exposure and cardiovascular diseases was controversial in a different study (26) and this discrepancy might be explained by the healthy worker effect (27).

From 2005-2006, lung cancer was the primary malignant disease of asbestos-related diseases in the study area. Most of the individuals engaged in the asbestos processing industry between 1970 and 1980, and the latency periods between asbestos exposure and disease range from 20-40 years [e.g., lung cancer (28)]. Lung cancer was the leading cause of cancer death in China between 2000 and 2011 (29). Previous work (30) among chrysotile asbestos textile workers in the USA reported increased mortality from lung cancer, owing to exposure to the long, thin chrysotile fibers. To our knowledge, the asbestos used in the asbestos processing industry in the study city was chrysotile (31). Therefore, our data supported the hypothesis that lung cancer mortality was strongly associated with exposure to the long, thin asbestos fibers.

Additionally, we found that SPMR for lung cancer was higher among females than males when compared at the same exposure levels. In particular, SPMR for lung cancer was significantly elevated among females who had experienced high levels of asbestos exposure. This result was similar to that reported in a previous study in Japan (10), which studied 143,929 residents in a city in Japan with many asbestos factories. We found SPMRs increased among females from low to high asbestos exposure levels, indicating an exposure-response relationship between lung cancer mortality and asbestos exposure levels. These results strongly supported the evidence that chrysotile exposure caused excess mortality due to lung cancer among women—even several decades after asbestos exposure cessation (32). More focus should be paid to the former female workers in this area, especially to the older women in this area.

We did not observe any mesothelioma cases in the death cause database from 2005 to 2006. This should be interpreted by the incomplete registry system of death causes and low diagnostic technology of mesothelioma in this study area. Moreover, mesothelioma may be misdiagnosed as lung cancer, ovarian cancer, or another malignancy (33). To this end, Weng and colleagues (34) reported the first case of mesothelioma in 1999 in the study city, which was consistent with our estimated data. The estimated mortality rate of mesothelioma was 2-6 per million in the study city; however, the global death rate of mesothelioma was 9.9 per million (35). Therefore, we believe that the mortality rate of mesothelioma was under-estimated in the study city during this period. Besides, a previous study in Italy (36) predicted that the peak of mesothelioma cases would reach 28-32 years after the ban on asbestos use. A similar study (37) using the distributed lag non-linear model showed that the risk of mesothelioma grew until 30 years after asbestos exposure and decreased thereafter. Hence, we inferred that lung cancer was the leading cause of death among asbestos-exposed individuals before 2006, while mortality due to mesothelioma would substantially increase in the study city after 2006 even after asbestos was banned in 2012. According to a national report in China, mesothelioma mortality rapidly increased across this time frame, having nearly doubled from 1990 to 2013 (22). Given this, significant attention will be needed to the future incidence of mesothelioma in this study city. There is still much to do to improve the pathological diagnosis ability of mesothelioma in the study area.

High mortality due to respiratory system diseases was confirmed with an elevated SPMR among asbestos-exposed females. Asbestosis may be the main reason for the mortality of respiratory system diseases among females. However, pneumoconiosis was not listed as a separate disease in the death certificates, so we did not have any pneumoconiosis data among the deaths caused by respiratory system diseases. Our stratified analysis also showed that mortality from respiratory system diseases increased with asbestos exposure levels among males. This result was consistent with a previous study conducted in China (38). Moreover, Naik and colleagues (21) found elevated mortality rates due to chronic obstructive pulmonary disease and asbestosis using death certificate data in the US state of Montana (SMR = 1.2 and 102.47, respectively). Moreover, a cohort study of chrysotile workers in Western China found a significant exposure-response relationship between asbestos exposure level and asbestosis risk (16). It is worth noting that the non-malignant asbestos-related diseases were closely correlated with the risk of malignant diseases (39). These findings further support the urgent need to prevent asbestos-related respiratory diseases and underscore the need for early diagnosis of malignant diseases among these non-malignant populations.

Our study has a number of strengths and some limitations. First, this is the first study to conduct a face-to-face investigation on causes of death in an asbestos processing area in China and a large sample of 2,964 death cases was selected for the study. Second, a dose-dependent relationship was found between asbestos exposure level and mortality of lung cancer and respiratory system diseases among women. There were also some limitations in this study. For instance, investigating information obtained from relatives of those individuals who had died might cause information bias. Repeatability of investigation results was found to decline with longer recall periods between the death and the interview. Therefore, the interval between the death and interview was suggested to be less than two years, in order to better control for recall bias and improve repeatability (40). In our study, well-trained investigators interviewed the relatives of those individuals who had died less than two years from death, thereby minimizing recall bias.

In summary, the current study on death cases confirmed the mortality pattern in the asbestos processing area of Eastern China. We detected a significantly increased mortality from lung cancer and respiratory system diseases in an asbestos processing area, particularly among women. The increased mortality from these causes of death was associated with chrysotile exposure. This study adds new understanding to the cause of death in an asbestos processing area in Eastern China. Our findings may potentially affect screening for lung cancer among women with a history of asbestos exposure.

## Data availability statement

The raw data supporting the conclusions of this article will be made available by the authors, upon reasonable request.

## Ethics statement

The studies involving human participants were reviewed and approved by the ethics committee of Zhejiang Academy of Medical Sciences, China. The patients/participants provided their written informed consent to participate in this study.

## Author contributions

Conceptualization: JL and XZ. Data curation: ZQJ. Formal analysis: ZQJ. Funding acquisition: XZ and ZJ. Investigation: JFC, JQC, LF, HZ, LJ, LZ, YX, ZYJ, CX, and DY. Methodology: JQC and ZQJ. Project administration: JL and XZ. Resources: DY. Software: MJ and ZQJ. Supervision: JL and XZ. Validation: MJ. Visualization: MJ and ZQJ. Roles/writing - original draft: ZQJ, JQC, and JL. Writing - review and editing: ZQJ and JL. All authors contributed to the article and approved the submitted version.

## Funding

This study was supported by grants from the Health Commission of Zhejiang Province (11-ZC02, 2019RC142), Key Projects of International Science and Technology Innovation Cooperation Between Governments, National Key R&D Plan (2019YFE0116100), and Hangzhou Medical College (KYYB202113).

## Conflict of interest

The authors declare that the research was conducted in the absence of any commercial or financial relationships that could be construed as a potential conflict of interest.

## Publisher’s note

All claims expressed in this article are solely those of the authors and do not necessarily represent those of their affiliated organizations, or those of the publisher, the editors and the reviewers. Any product that may be evaluated in this article, or claim that may be made by its manufacturer, is not guaranteed or endorsed by the publisher.
