# Supplementary material for: Mortality due to respiratory system disease and lung cancer among female workers exposed to chrysotile in Eastern China: A cross-sectional study
Source: Front Oncol. 2022 Aug 1;12:928839. doi: 10.3389/fonc.2022.928839 (PMC9376437; doi:10.3389/fonc.2022.928839)
Supplement: Supplementary file 1 [file Table_1.docx]

Supplementary Table 1. Observed deaths and expected deaths by gender and occupational asbestos exposure

| Diseases | Occupational asbestos exposure | | No occupational asbestos exposure | |
| --- | --- | --- | --- | --- |
|  | Observed | Expected | Observed | Expected |
| **Male** |  |  |  |  |
| Respiratory system diseases | 3 | 1.24 | 68 | 88.66 |
| Circulatory system diseases | 3 | 4.30 | 137 | 295.46 |
| Lung cancer | 0 | 1.44 | 83 | 89.36 |
| Liver cancer | 1 | 0.81 | 68 | 57.37 |
| Gastric cancer | 0 | 0.66 | 56 | 41.58 |
| Esophageal cancer | 0 | 0.32 | 21 | 20.63 |
| Cancer of colon, rectum and anus | 0 | 0.31 | 6 | 21.02 |
| Other malignant tumors | 1 | 0.93 | 32 | 64.87 |
| Other diseases | 2 | 1.98 | 112 | 170.06 |
| All cancer | 2 | 4.47 | 266 | 294.82 |
| **Female** |  |  |  |  |
| Respiratory system diseases | 4 | 3.32 | 39 | 45.58 |
| Circulatory system diseases | 10 | 13.50 | 85 | 168.67 |
| Lung cancer | 1 | 3.12 | 37 | 34.52 |
| Liver cancer | 3 | 1.35 | 35 | 14.43 |
| Gastric cancer | 0 | 1.16 | 14 | 13.79 |
| Esophageal cancer | 0 | 0.38 | 2 | 4.42 |
| Cancer of colon, rectum and anus | 0 | 1.12 | 5 | 12.36 |
| Other malignant tumors | 3 | 5.06 | 34 | 58.54 |
| Other diseases | 8 | 7.01 | 66 | 91.68 |
| All cancer | 7 | 12.18 | 127 | 138.06 |
